# The Convalescent Homes Association

**Published:** 1905-07-15

**Authors:** 


### HOSPITAL ADMINISTRATION.

### CONSTRUCTION AND ECONOMICS.

### The Convalescent Homes Association.

Modern charitable efforts in dealing with the sick are recognising with increasing conviction the importance of two commanding general principles. The first of these is the necessity for systematic co-ordination and co-operation on sound business lines, and the second, the great enlargement of the scheme of hospital relief rendered possible by the establishment of the convalescent home. It is this latter development which, in addition to its direct benediction to individuals, has increased greatly the ability of the hospitals to meet the demands made upon them. For the most part the convalescent home has arisen under the pressure or accident of circumstance. In many instances it has been the outcome of a modest personal effort aroused by sympathy with suffering. Hence it is not surprising that these institutions have developed without the direction of any general plan, and free from the regulation of a considered policy. Their relationship to the public and to the hospitals is in many cases an ill-defined one, and their isolation and detachment prevent the accumulation of a common experience for their mutual benefit. It is fitting that an effort should be made to improve this state of affairs, and to arrange a plan and order worthy of the importance of the position which the convalescent home now occupies in the scheme of charitable relief. With these ends in view, it has been determined to establish a Convalescent Homes Association, and a meeting to promote this object was held at 20 Hanover Square, W., on the 6th inst. under the chairmanship of Sir E. Hay Currie.

The declared objects of the new association are to co-ordinate the work of public convalescent homes receiving London convalescent patients; to obtain and disseminate information on the needs of London in respect of convalescent relief; to advise convalescent institutions on matters of equipment, management, and maintenance; to protect their rights and interests, and to promote communication and co-operation between convalescent homes on the one hand, and hospitals and other public institutions on the other. These objects are certainly well adjusted to the demands of the situation, and so far as can at present be judged, the proposed constitution is likely to lead to a successful issue. Experience will, no doubt, reveal opportunities for improvements and for further developments, but the great gain is that the possibility of such advances will be secured to all the institutions concerned by the establishment of a representative body where common purposes and common difficulties can be discussed. It is on this arrangement that confidence in the new Association can be securely based. It may confidently be expected to bring order, efficiency, and systematised usefulness into a department of charitable work in which at present isolation and absence of organisation are prominent features.

The suggestion is that each home joining the Association shall contribute to a central fund and shall have one or more representatives on the Council in proportion to the number of beds it provides. In addition, any metropolitan hospital contributing according to its size to the funds of the Association will be entitled to send a representative to the Council, on which, also, the Hospital Sunday and Saturday Funds, the Central Hospital Council, the Charity Organisation Society, and the Invalid Children's Aid Association will each have a representative. It is further provided that the Presidents of the Royal College of Physicians and the Royal College of Surgeons shall be *ex officio* Vice-Presidents of the Association.

These arrangements can hardly fail to secure a proper representation of all the interests concerned and are likely, we think, also to command public confidence. It is satisfactory to see that the Association will only recognise those homes which are genuine charitable institutions, and which publish reports (we presume annual reports) of their income and expenditure. Certain institutions of loud charitable profession will in this way be excluded, and thus, it is to be hoped, will gradually be reduced in the public estimate to their proper position. If we might venture to suggest a danger from the new Association, it would be the risk of an undue desire for conformity to an official plan and pattern. More or less this chance pertains to all central organisation. But it will doubtless be remembered that convalescent homes have to satisfy very different conditions in different cases, and that there is need not only for the large and ordered institution with its many activities, but also for the quiet and secluded retreat and resting-place. The necessary rules and discipline of the one have their place and value, but equally so have the ease and relaxation of the other. For ourselves, we offer the Association our cordial goodwill, and confidently anticipate for it a large sphere of usefulness. The honorary secretaries are M. O. Fitzgerald, Esq., 130 Mount Street, W., and Dr. Habershon, 88 Harley Street, W.

